# *SPIND*: a reference-based auto-indexing algorithm for sparse serial crystallography data

**DOI:** 10.1107/S2052252518014951

**Published:** 2019-01-01

**Authors:** Chufeng Li, Xuanxuan Li, Richard Kirian, John C. H. Spence, Haiguang Liu, Nadia A. Zatsepin

**Affiliations:** a Department of Physics, Arizona State University, Tempe, Arizona 85287, USA; b Center for Applied Structural Discovery, The Biodesign Institute, Arizona State University, Tempe, Arizona 85287, USA; c Complex Systems Division, Beijing Computational Science Research Center, Beijing, 100193, People’s Republic of China; d Department of Engineering Physics, Tsinghua University, Beijing, 100086, People’s Republic of China

**Keywords:** serial crystallography, X-ray free-electron lasers, XFEL, electron diffraction, diffract-then-destroy, dynamical studies, auto-indexing algorithms, Bragg peaks

## Abstract

*SPIND* (sparse-pattern indexing) is an auto-indexing algorithm for sparse snapshot diffraction patterns (‘stills’) that requires the positions of only five Bragg peaks in a single pattern, when provided with unit-cell parameters. The capability of *SPIND* is demonstrated for the orientation determination of sparse diffraction patterns using simulated data from microcrystals of a small inorganic molecule containing three iodines, 5-amino-2,4,6-triiodoisophthalic acid monohydrate (I3C) [Beck & Sheldrick (2008 ▸), *Acta Cryst.* E**64**, o1286], which is challenging for commonly used indexing algorithms. *SPIND*, integrated with *CrystFEL* [White *et al.* (2012 ▸), *J. Appl. Cryst.* **45**, 335–341], is then shown to improve the indexing rate and quality of merged serial femtosecond crystallography data from two membrane proteins, the human δ-opioid receptor in complex with a bi-functional peptide ligand DIPP-NH_2_ and the NTQ chloride-pumping rhodopsin (CIR). The study demonstrates the suitability of *SPIND* for indexing sparse inorganic crystal data with smaller unit cells, and for improving the quality of serial femtosecond protein crystallography data, significantly reducing the amount of sample and beam time required by making better use of limited data sets. *SPIND* is written in Python and is publicly available under the GNU General Public License from https://github.com/LiuLab-CSRC/SPIND.

## Abstract 2

*SPIND* is an auto-indexing algorithm developed for sparse-pattern diffraction data. It is generally applicable to multiple crystallographic data types including serial crystallographic data collected from X-ray free-electron lasers (XFEL), synchrotron light sources or transmission electron diffraction data collected on a transmission electron microscope (TEM), and can also be extended to ultrafast electron diffraction data. The algorithm and comparative studies on experimental serial femtosecond protein crystallography data sets demonstrate its ability to maximize data efficiency by making use of sparse patterns that are discarded by other indexing programs.

## 1. Introduction   

The high brightness and femtosecond pulse duration of X-ray free-electron lasers (XFELs) enabled the serial diffraction-before-destruction paradigm (Neutze *et al.*, 2000 ▸), which mitigates X-ray radiation damage and allows data to be collected from weakly scattering targets. In a typical serial femtosecond crystallography (SFX) experiment, diffraction patterns are recorded from tens of thousands of microcrystals delivered sequentially across a pulsed X-ray beam [Chapman *et al.* (2011 ▸); see Spence (2017 ▸) for a review]. These snapshot diffraction patterns (from individual microcrystals) correspond to reciprocal-space intensity samples that lie on the surface of the Ewald sphere. Since each crystal is in a random orientation, crystal orientations must be determined before intensities can be merged in three-dimensional reciprocal space. Femtosecond XFEL pulses are too short for substantial crystal rotation during exposure, so only partial reflection intensities are recorded in each diffraction pattern, with partiality determined by various factors such as X-ray bandwidth and crystal shape, size, orientation and mosaicity.

SFX data analysis is challenging because of the wide variation in crystal size and mosaicity, which is confounded by jitter in the XFEL pulse energy and spectrum, detector dynamic range limitations, and the random positions/orientations of crystals. Monte Carlo integration (Kirian *et al.*, 2010 ▸) is an effective means of producing crystallographic structure factors by simply averaging over stochastic measurement variations that are assumed to be completely random. However, this approach requires a large number of patterns (on the order of tens of thousands) in order for the average partial reflection measurements to converge to a reliable set of integrated Bragg intensities [see Li *et al.* (2015 ▸) for error metric analysis of the Monte Carlo integration approach]. This is in contrast to conventional synchrotron crystallography in which the molecular structure is determined using one or a few larger crystals, using the oscillation approach where the crystals are rotated through the Bragg condition during the intensity recording to yield angle-integrated structure factors. Post-refinement techniques for SFX data have recently been developed that can greatly reduce the number of required snapshot patterns to a few thousand, or a few hundred, in favorable circumstances (White, 2014 ▸; Uervirojnangkoorn *et al.*, 2015 ▸; Sauter, 2015 ▸; Ginn *et al.*, 2015 ▸). This reduces demand on scarce XFEL beam time and sample volume.

A number of software packages have been developed specifically for SFX or snapshot diffraction data analysis [see Liu & Spence (2016 ▸) for a review]. An SFX data-analysis pipeline commonly starts with data-reduction programs such as *Cheetah* (Barty *et al.*, 2014 ▸), which apply various detector calibrations, identify diffraction peaks and produce data-collection statistics. Patterns containing diffraction peaks are then passed to programs such as *CrystFEL* (White *et al.*, 2012 ▸) or *cctbx.xfel* (Hattne *et al.*, 2014 ▸) for high-throughput auto-indexing and intensity merging. *CrystFEL*’s program *indexamajig* calls subroutines wherein partial reflections are auto-indexed and locally integrated within each two-dimensional pattern. Finally, intensities from partial reflections are merged by *process_hkl* (optionally including scaling and post-refinement using *partialator*), which results in a set of Bragg intensities. For each diffraction pattern, *indexamajig* passes the peak positions as input arguments to auto-indexers such as *MOSFLM* (Powell, 1999 ▸), *DirAx* (Duisenberg, 1992 ▸), or *XDS* (Kabsch, 1988 ▸, 1993 ▸), or algorithms implemented directly in *indexamajig*, such as *asdf, felix* (Beyerlein *et al.*, 2017 ▸) and *taketwo* (Ginn *et al.*, 2016 ▸). Auto-indexing modules return a set of lattice vectors oriented in the laboratory frame and work by first converting Bragg reflections to three-dimensional reciprocal-space vectors. *MOSFLM* or *LABELIT*, for example, then projects these vectors onto a set of discrete directions distributed about a hemisphere (Campbell, 1998 ▸; Leslie, 2006 ▸; Powell, 1999 ▸; Steller *et al.*, 1997 ▸; Sauter *et al.*, 2004 ▸). If a sufficient number of diffraction peaks from a single crystal lattice contribute to the one-dimensional projected histogram, its Fourier transform consists of sharp spikes if the projection direction coincides with one of the principal axes of the crystal. The frequency of these spikes provides lattice parameter information. Once principal axis candidates are identified, *indexamajig* predicts the possible Bragg peak positions in the original pattern, tests for reasonable agreement with the observed peak positions, and if the agreement is satisfactory, the peak intensities are integrated. The result of this procedure is a set of partially integrated reflection intensities and associated Miller indices.

This data-analysis pipeline has been used for high-resolution structure determination in both SFX and synchrotron serial crystallography (Nogly *et al.*, 2016 ▸; Standfuss & Spence, 2017 ▸). However, a significant portion of SFX diffraction patterns, especially those from difficult-to-crystallize macromolecules such as membrane proteins, only have a small number of identifiable Bragg peaks because of small crystal size, crystal disorder, scattering from air and sample-delivery medium, and detector noise, which decrease the signal-to-noise ratio (SNR). Time-resolved SFX requires small crystals because the extinction length of the optical-pump laser is typically on the order of 10 µm, while the temporal resolution of mix-and-inject SFX (to study ligand binding, for example) is limited by mixing and diffusion rates, and hence also benefits from smaller crystal size (Schmidt, 2013 ▸). The narrow (though spiky) bandwidth of XFELs based on self-amplified spontaneous emission (SASE) and the stillness of crystals during the ultrafast exposures in SFX also limits the number of Bragg spots that intersect the Ewald sphere. In addition, inorganic crystals typically have very small unit cells and so give rise to sparse patterns with fewer Bragg peaks in the same resolution shell than most protein crystals. Existing auto-indexing algorithms that are based on one-dimensional Fourier transforms typically require that each pattern consist of 20 [or even more (Campbell, 1998 ▸)] accurately identified Bragg peaks to yield a reliable crystal orientation. The development of auto-indexing algorithms for sparse patterns with fewer peaks would greatly increase SFX data utilization for such challenging cases with low resolution, and for samples with very small unit cells. Here we take sparse to mean that there are few Bragg spots, rather than weak Bragg intensities.

In this paper, we present a new algorithm, *SPIND* (sparse-pattern indexing), designed to index patterns with sparse data, achieve faster and more accurate structure-factor measurements, and reduce measurement time, sample consumption and cost. The use of angles between scattering vectors, as well as their lengths, is a strong constraint, as described in *Methods*. *SPIND* has the merit of a low false-positive rate and hence a high level of effectiveness as well as efficiency, which is demonstrated on extremely sparse patterns simulated from inorganic crystals and experimental SFX data from membrane-protein microcrystals. Two alternative auto-indexing algorithms for sparse patterns have been developed recently. Maia *et al.* (2011 ▸) developed a compressive sensing-based auto-indexing algorithm for sparse diffraction patterns in serial femtosecond nanocrystallography in which lattice reconstruction is reformulated as an L1 minimization (basis pursuit) problem. The algorithm was shown to efficiently reconstruct a three-dimensional lattice and its orientation from a simulated noise-free sparse diffraction pattern without prior knowledge of the unit cell. The use of multiple three-dimensional fast Fourier transforms renders the algorithm computationally expensive in its current form, but incorporating additional algorithms designed for sparse data should substantially increase its speed. Additionally, the indexing ambiguity arising from mirror symmetries of the lattice remains to be resolved, and the algorithm is yet to be demonstrated on experimental data or in the presence of noise. An alternative auto-indexing algorithm for sparse SFX diffraction patterns from crystals with small unit cells, which depends on known lattice parameters, was demonstrated by Brewster *et al.* (2015 ▸) on amyloid peptide nanocrystal data. The approach consists of three steps: (1) assign each peak all possible Miller indices corresponding to its resolution, (2) resolve the ambiguities in Miller-index assignment (*e.g.* from lattice symmetry or semi-overlapping powder rings) and (3) calculate basis vectors and refine crystal orientation, iteratively. The Bron–Kerbosch algorithm (Cazals & Karande, 2008 ▸) is used to determine the maximum-clique of a graph in which all found peaks, with each of their candidate Miller indices, are represented as individual nodes. For each pair of peaks, the differences between calculated and observed inter-peak distances in reciprocal space (for each candidate Miller index) are represented as edges, so Bragg peaks that cannot be simultaneously accounted for by one orientation matrix are not connected in the graph. An advantage of the graphic maximum-clique algorithm is its tolerance to false peaks, but for very sparse patterns (five Bragg peaks), the determined unit-cell accuracy is on the order of 10%.

## 2. Methods   

**Figure 1:**
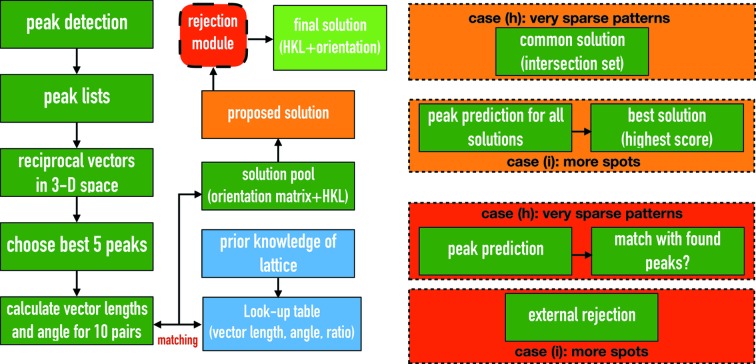
Flowchart of the *SPIND* indexing algorithm. The five best peaks in each pattern selected based on user-chosen criteria, such as SNR, are used for indexing. The blue boxes refer to prior knowledge and the table is calculated once. The green boxes are steps carried out for each pattern. The (red) rejection module refers to steps (*g*)[Other li1g] to (*i*)[Other li1i].

**Figure 2:**
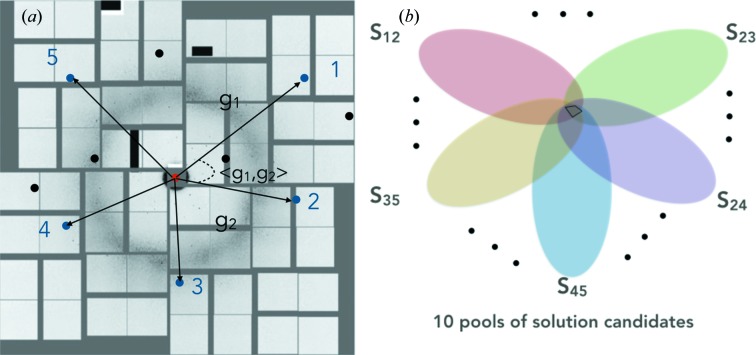
Illustration for *SPIND* auto-indexing algorithm. (*a*) A diffraction pattern recorded by Cornell-SLAC hybrid Pixel Array Detector (CSPAD) (Herrmann *et al.*, 2013 ▸; Hart *et al.*, 2012 ▸), with a few exaggerated peaks for illustrative purposes. Five peaks are selected to form ten vector pairs. The vector lengths, ratio of lengths and angles between the vectors are then calculated for the ten pairs for matching with a reference based on *a priori* knowledge of the unit cell (within some mismatch tolerance). (*b*) Rejection module for eliminating spurious solution candidates, based on the constraint that all peak pairs share the same crystal orientation. The solution must lie in the intersection of the solution pools, provided that the peaks are from a single crystal.

Here we describe the algorithm for indexing patterns containing very few Bragg peaks, which we refer to as *SPIND*. If peaks are sparsely distributed in each pattern, their periodicity is difficult to identify *via* Fourier methods. The proposed algorithm therefore utilizes prior knowledge of unit-cell parameters. The algorithm works as follows (see Fig. 1 ▸ for a flowchart). For each pattern:

1. (*a*) Bragg peaks are identified and their positions, intensities, SNRs, resolution and the camera length are written to a plain text file.
2. (*b*) The two-dimensional peak positions on the detector are converted to three-dimensional reciprocal vectors that start from the beam center (treated as a 000 reflection) using the experimental geometry.
3. (*c*) The best five peaks (or more) are chosen from the peak list according to intensity, SNR or resolution (these can be chosen by the user). In principle, any number of peak pairs greater than two can be used to determine the crystal orientations. Indexing reliability increases with the number of pairs used, as discussed later. However, the computation time scales roughly as *N* ^2^ {the number of pairs scales as 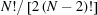 where *N* is the number of peaks}. We found that five peaks (ten peak pairs) is a good compromise between indexing yield and computational time.
4. (*d*) For each of the ten pairs, the two vector lengths and angle between the pair are calculated [see Fig. 2 ▸(*a*)].
5. (*e*) For a large set of discretized crystal orientations, a reference table is created that contains the expected lengths, ratio of the lengths, and angles between vectors for all possible pairs (within a certain resolution limit). This reference table is based on prior knowledge of the unit cell and is calculated only once in the whole process of indexing.
6. ( *f* ) The set of observed vector lengths, ratios of lengths (in case the geometry needs refinement), and angles corresponding to each of the ten pairs of peaks are compared with the entries in the reference table. Whenever a match is found between a given observed peak pair and an entry in the reference table (within a preset mismatch tolerance), the reference-table entry (*i.e.* crystal orientation) is considered to be a solution candidate and added to a solution pool. One solution pool, with multiple entries, is created for each of the ten peak pairs.
7. (*g*) If all reciprocal vectors correspond to the same crystal orientation, the true solution for the crystal orientation must be in the intersection of all ten solution pools (Fig. 2 ▸ *b*). This is a strong constraint that effectively eliminates spurious solution candidates in one step without implementing other optimization and clustering algorithms such as in the work by Brewster *et al.* (2015 ▸). However, this constraint is sensitive to peaks not belonging to a single lattice, which will require development of approaches for eliminating false peaks used for indexing. The indexing ambiguity that occurs in cases where the symmetry of Bravais lattice is higher than the space-group symmetry is not resolved here. Several algorithms have been designed and tested to resolve this type of indexing-ambiguity problem, in particular for serial crystallography data (Brehm & Diederichs, 2014 ▸; Liu & Spence, 2014 ▸).
8. (*h*) For very sparse patterns, if a single orientation is found in the intersection, it is used to predict peak locations. If the distance between predicted and found peaks of the same Miller index (in three-dimensional reciprocal space) is within the tolerance threshold, the peaks are tagged as matched. By default, this threshold is half of the distance to the nearest Bragg peak. If the predicted peaks match all found peaks in the experimental pattern, the solution is accepted; otherwise, the pattern is considered un-indexable and rejected.
9. (*i*) For diffraction patterns containing more spots: all candidate orientations (from all solution pools) are used to predict peak locations. The quality of the orientation candidate solution is scored by the number of predicted peaks matching observed peaks (for all peaks, not just those used for indexing), the percentage of peaks matched (a higher percentage of matched peaks is required if the total number of peaks is low) and the determined lattice centering.

## 3. Results and discussions   

### 3.1. Simulating and indexing sparse patterns from inorganic microcrystals   

**Figure 3:**
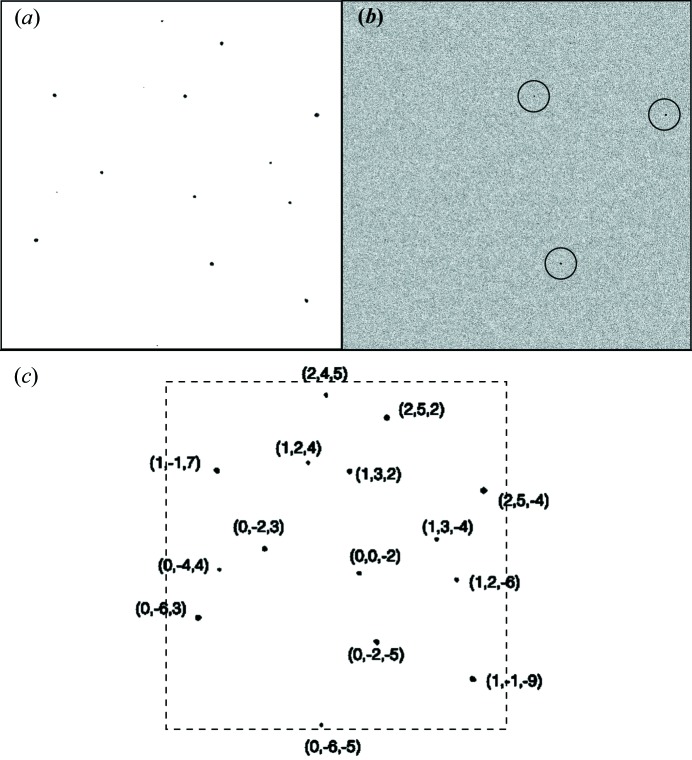
Simulated I3C patterns indexed by *SPIND*. (*a*) Simulated sparse diffraction pattern from an I3C crystal in the orientation specified by Euler angles −10.4676, 46.9022, 139.1443. (*b*) Poisson noise and random background noise added to (*a*), so only three Bragg peaks were identifiable (circled). (*c*) The indexing result by *SPIND* using only the three peaks in (*b*). The determined crystal orientation is at Euler angles of −10.5713, 46.8855, 139.2000. The peaks were predicted from the determined orientation and Miller indices were given for Bragg peaks.

In order to test the ability of *SPIND* to index sparse diffraction patterns, we simulated 400 diffraction patterns from 5-amino-2,4,6-triiodoisophthalic acid monohydrate (I3C) crystals (Beck & Sheldrick, 2008 ▸) at random orientations. A unit cell with *a* = 9.02, *b* = 15.73, *c* = 18.82 Å, α = β = γ = 90°, a photon energy of 9.61 keV, 0.5 µm beam radius, 110 × 110 µm^2^ detector pixel size, and 53.2° maximum scattering angle at a working distance of 0.07 m were used for diffraction-pattern simulations. The three-dimensional profile of the reciprocal lattice points was modeled as Gaussians taking into account 1 µm crystal size and structure factors. The intensities were calculated from the three-dimensional Gaussian profile and the excitation error, defined as the distance between the Ewald sphere (corresponding to monochromatic X-ray beam of 9.61 keV photon energy) and the center of the reciprocal lattice point projected in the beam direction. Poisson noise and background scattering were included such that only three to five Bragg peaks were identifiable in each pattern [see Figs. 3 ▸(*a*) and 3(*b*) for a representative pattern before and after adding noise terms]. The *SPIND* algorithm obtained correct crystal orientations and Miller indices for all 400 patterns. This implementation of *SPIND* (written in *MATLAB* R2014b; The MathWorks Inc., Natick, MA, USA) required millisecond computational time on a Mac 2.7 GHz Intel Core i7. In addition to the fast computation time, the orientation was determined with high accuracy. A typical example from the 400 patterns that were indexed successfully is shown in Fig. 3 ▸(*c*), where the orientation was determined with an accuracy of around 0.1°.

**Figure 4:**
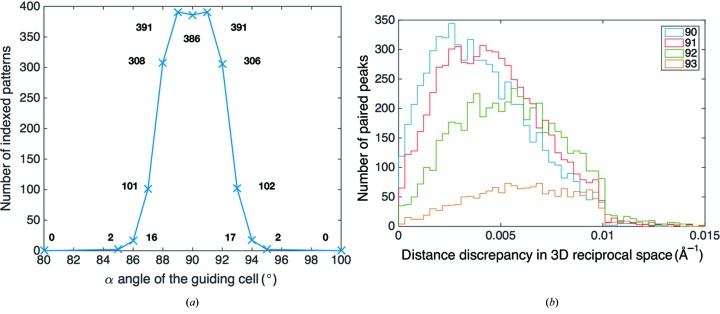
The effect of inaccurate guiding unit cells on *SPIND* indexing rates and peak-prediction accuracy demonstrated on simulated I3C snapshot diffraction patterns. (*a*) Number of indexed patterns as a function of the α angle of the guiding cell (α = 90° is nominal). (*b*) Distribution of distance discrepancy in three-dimensional reciprocal space between found and predicted peaks for matched peak pairs using guiding cells with different α angle values. The legend shows the α angle of the guiding cell. The center of the distribution shifts to larger values as α deviates further from the nominal value of 90°. The same trend was observed for values of α < 90° (omitted for clarity). The results demonstrate the robustness of the algorithm to the lattice inhomogeneity, a wide tolerance range for the guiding-cell constants and low false-positive indexing rate when the target lattice cell is clearly distinguishable from the guiding cell. The indexing rate can be used as an indicator for the accuracy of the reference unit cell.

To investigate the robustness of the indexing algorithm in the presence of lattice inhomogeneity or inaccurate guiding-cell constants, an additional set of 400 I3C diffraction patterns was simulated with random Gaussian fluctuations in lattice constants about the mean values with 0.5% standard deviation. *SPIND* indexing was carried out using 11 different guiding unit cells with varying α angle values and lengths of *b* and *c* basis vectors. The α angle of the guiding cell was varied from 80 to 110° symmetrically around the nominal value of 90°, with the length of the *b* and *c* basis vectors adapted such that the volume of the cell is invariant (same as the nominal cell used to simulate the diffraction patterns) to maintain a consistent average density of the Bragg orders as a control factor. The number of indexed patterns decreased as the guiding cell deviated incrementally from the nominal cell (Fig. 4 ▸ *a*). In this test, the peak indexing rate appears at the α angle values of 89 and 91°, which is most likely attributable to the random fluctuations in lattice constants combined with the limited size of the diffraction data set. To validate this argument, the distribution of the three-dimensional reciprocal-space distances between the predicted and the found positions for the paired peaks from all indexed patterns were obtained for the α angle values of 90, 91, 92 and 93° (Fig. 4 ▸ *b*). The most probable value of the distance between the predicted and the observed peak positions is minimized when α = 90° and increases consistently as the α value incrementally deviates from 90°. In addition, the total number of paired peaks also drops as the guiding cell deviates from the nominal. In accordance with the symmetry of the orthorhombic I3C lattice, the indexing rate and distance discrepancy for paired peaks are also essentially symmetric about α = 90°. For this reason, only half of the distribution statistics, corresponding to α > 90°, are shown in Fig. 4 ▸(*b*) for visual clarity. The abrupt drops of the paired-peak population at 0.01 Å^−1^ in Fig. 4 ▸(*b*) arise from this same cut-off value set for the peak-match judgment [step (*h*)[Other li1h] in the *Methods* section]. The indexing rate drops to approximately 25% at α = 87, 93° which shows a ±3° tolerance range for the uncertainty or inaccuracy in the guiding-cell constants. The indexing rate drops rapidly further to 0.5% at α = 85, 95° and goes to 0 beyond 10° of deviation. This low false-positive indexing rate verifies the reliability of the *SPIND* rejection module. These indexing statistics using guiding cells that deviate incrementally from the nominal have demonstrated the robustness of the *SPIND* indexing algorithm to the lattice inhomogeneity, a wide tolerance range for the guiding-cell constants and low false-positive indexing rate when the target lattice cell is clearly distinguishable from the guiding cell. Furthermore, it is worthwhile to point out that the guiding cell, as well as other indexing parameters such as error tolerance and threshold values (see the *Methods* section[Sec sec2]), can be updated iteratively by using indexing statistics (see Fig. 4 ▸ as an example) as feedback. Individual pattern-based lattice refinement, together with this iterative updating of the guiding cell and indexing parameters are part of ongoing development.

### 3.2. Indexing SFX data from a G-protein-coupled receptor complex   

To demonstrate the algorithm on protein serial crystallography data, *SPIND* was used to index serial X-ray diffraction data from microcrystals of a G-protein-coupled receptor (GPCR) complex, the human δ-opioid receptor in complex with a bi-functional peptide ligand DIPP-NH2 (referred to as DOR henceforth) collected at the Coherent X-ray Imaging (CXI) endstation of the Linac Coherent Light Source (LCLS) (Fenalti *et al.*, 2015 ▸; Liang *et al.*, 2015 ▸).

We used the diffraction patterns from the data set ID 40 in the Coherent X-ray Imaging Data Bank [CXIDB, Maia (2012 ▸)]. For CXIDB 40, the LCLS raw data, containing over 1 967 530 detector frames, were reduced using *Cheetah* for hit finding, leaving 125 458 diffraction patterns from DOR microcrystals. Indexing and intensity integration were performed with *CrystFEL* 0.6.2 (White, Barty *et al.*, 2016 ▸), based on the same *indexamajig* and *partialator* parameters as used for the original processing (Fenalti *et al.*, 2015 ▸). The indexing was attempted by *MOSFLM* 7.2.0 (using prior unit-cell parameters and lattice type information), followed by *DirAx*, and finally *MOSFLM* without any prior information. For a fair comparison of the indexing rate and accuracy of *SPIND* on SFX data, the DOR data were reprocessed using one indexing method at a time rather than combinations thereof [the latter was the approach in the work by Fenalti *et al.* (2015 ▸) and (White, Barty *et al.*, 2016 ▸). The auto-indexing methods that are compared in this work are *SPIND*, *DirAx*, and *MOSFLM* (with and without lattice type and unit-cell input). Also, the refinement option was toggled on and off in *indexamajig* to investigate the effect of the pattern filtering after *SPIND* auto-indexing on the quality of merged data.

The data processing was performed using the scripts deposited in CXIDB ID 40 with minimal necessary changes to keep the consistency for comparison between different indexing methods. A slightly larger unit cell than the published one was found to give more symmetric unit-cell distributions (see Fig. S1 in the Supporting information) and higher indexing rates, so the updated unit cell was used for all DOR indexing tests, with *a* = 160.10, *b* = 91.64, *c* = 99.05 Å, β = 92.22°. The other parameters used for data processing in this work are summarized in Tables 1 ▸ and 2 ▸. In general, the number of indexed patterns using *SPIND* algorithm increases with the resolution limit that the reference table is generated to [step (*e*)[Other li1e] in the *Methods*]. However, increasing the resolution limit of the reference table also results in longer computing time and higher demands on memory. Therefore, an optimal resolution limit of 8 Å was chosen after several trials to balance between the indexing rate and the computation time. Similarly, the tolerance threshold for reciprocal-vector search [step ( *f* )[Other li1f]] was set to be 3 × 10^7^ m^−1^ and 3° based on the performance of several trials.

**Figure 5:**
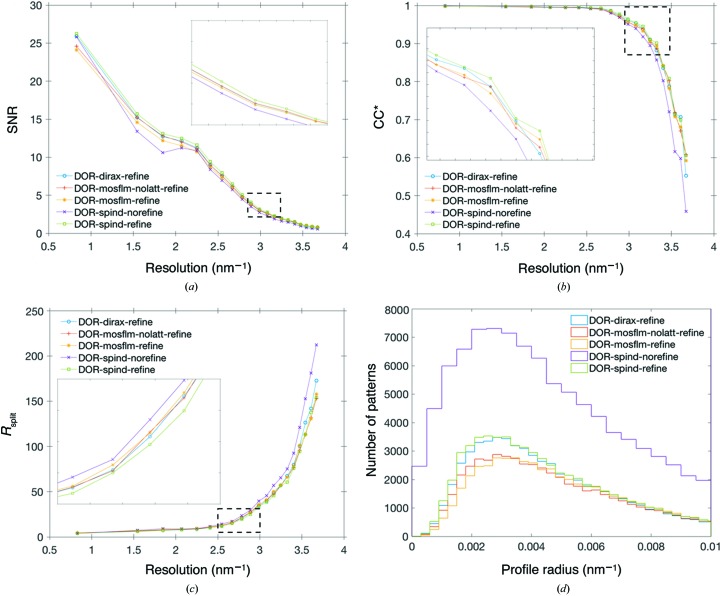
Figures-of-merit as a function of resolution for DOR SFX data. (*a*) SNR, (*b*) CC^*^, (*c*) *R*_split_ and (*d*) Bragg reflection profile radii determined by *indexamajig*. See Fig. S2(*a*) for full range of reflection profile radii and Fig. S2(*b*) for reflection multiplicity in merged data sets in the Supporting information. The keywords ‘refine’ and ‘norefine’ represent the on and off status of the lattice-refinement option in *indexamajig* in the indexing process. ‘nolatt’ represents that the reference cell and lattice type were not used as input for indexing (but were used as constraints for the indexing solution).

**Figure 6:**
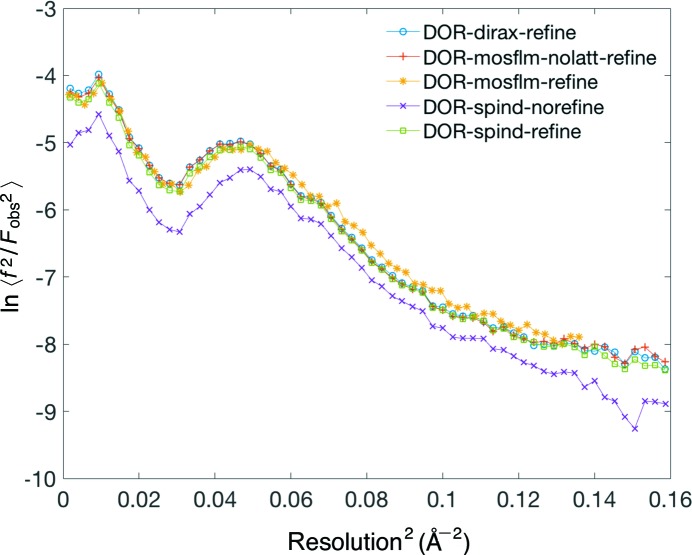
Wilson plots for the merged DOR data sets from different indexing methods. The linearity in the 0.06 to ∼0.13 Å^−2^ region and the consistency between all indexing methods confirm the quality of the merged data sets.

To evaluate the performance of *SPIND* alongside other indexing methods, the merged data quality metrics – SNR, multiplicity, and two metrics of data precision: CC* (Karplus & Diederichs, 2012 ▸) and *R*_split_ (White, Kirian *et al.*, 2012 ▸) – are summarized in Table 3 ▸ and plotted in Fig. 5 ▸. Compared with *MOSFLM* or *DirAx* individually, *SPIND* yielded the highest indexing rate (53.5%) with the orientation refinement option on in *indexamajig* (see Table 3 ▸), and 94.4% without the refinement. The refinement module in *indexamajig* of *CrystFEL* acts as a filter for the indexed patterns (White, Mariani *et al.*, 2016 ▸) by conserving only the patterns where the predicted peaks match the observed peaks sufficiently well while discarding the others, resulting in a reduced indexing rate. The multiplicity from *DirAx*-refine is slightly higher than that from *SPIND*-refine, while the overall SNR is lower (Fig. 5 ▸). *SPIND*-refine has the highest overall figures-of-merit – SNR, *R*_split_, and CC^*^ (Karplus & Diederichs, 2012 ▸) – in all resolution shells. These self-consistency figures-of-merit along with the Wilson plots (Fig. 6 ▸) for the merged data sets from different indexing methods verify the general reliability of the *SPIND* indexing algorithm and its applicability to serial protein crystallography data processing. (Otherwise, the figures-of-merit would become worse as patterns that are incorrectly indexed are merged towards the structure-factor list.) The *B* factors [calculated by *TRUNCATE* in *CCP*4 (French & Wilson, 1978 ▸)] were between 48 Å^2^ (*MOSFLM*-nolatt-refine) and 60 Å^2^ (*SPIND*-norefine). Moreover, the additional patterns indexed using *SPIND* and the improved figures-of-merit indicate the potential capability of mining more data for structure determination, and hence improving the data efficiency, by including this new algorithm in the serial crystallography data analysis routine.

By enabling indexing of patterns with much fewer peaks, *SPIND*-norefine yields much higher multiplicity [see Fig. S2(*b*) in the Supporting information], at the cost of other figures-of-merit (Fig. 5 ▸). The Bragg reflection profile radius (for each crystal) calculated by *indexamajig* is a measure of the excitation errors of matched peaks and thus serves as a measure of the accuracy of the determined orientation. The modal value of the reflection profile radii when using *SPIND*-norefine is the same as for the other indexing methods (Fig. 5 ▸ *d*), indicating *SPIND* orientation determination is sufficiently accurate for these patterns without the refinement module in *indexamajig*. However, 10% of the patterns had an insufficient number of matched peaks as determined by *indexamajig*, and the reflection profile radius was not updated from the default value of 0.02  × 10^9^ m^−1^ [see Fig. S2(*a*) in the Supporting information]. Removal of these patterns improved the SNR (and the CC*, marginally) in lower-resolution bins. The majority of DOR patterns were of lower resolution (see Fig. S3 in the Supporting information), and *SPIND*-norefine was able to index a larger portion of these [see Fig. S3( *f* ) in the Supporting information], with high accuracy indicated by the small reflection profile radii [see Fig. S4(*b*) in the Supporting information]. It is possible the lower overall quality of the merged *SPIND*-norefine data set is caused by the inclusion of potentially anisomorphous crystals (from the lower-resolution crystal batch), or lack of orientation and unit-cell refinement.

### 3.3. Indexing SFX data from chloride ion-pumping rhodopsin microcrystals   

Serial crystallographic data were collected from chloride-pumping rhodopsin (ClR) microcrystals at CXI, LCLS. Over 1 200 000 raw frames were collected in about 3 h. *Cheetah* identified 105 050 patterns as crystal hits with at least ten peaks per pattern with SNR > 8. After several rounds of geometry and lattice-cell refinement, 3414 patterns were indexed using *CrystFEL*, giving an indexing rate of approximately 3%, with a monoclinic unit cell where *a* = 103.45, *b =* 50.28, *c* = 69.38 Å, and β = 109.7°. Merging these 3414 indexed patterns led to a structure solution (based on molecular replacement) but with low figures-of-merit. This was attributed to the small number of indexed diffraction patterns (especially for a membrane protein) and poor diffraction quality in the higher-resolution range (the average multiplicity drops below 15 for resolution greater than 6 Å).

**Figure 7:**
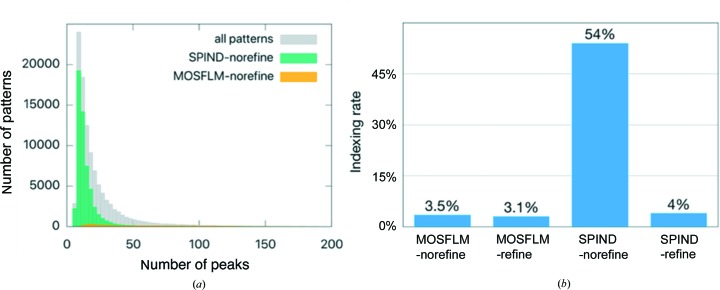
Statistics of ClR data set. (*a*) Distribution of number of peaks per pattern. Most patterns contained ten to 30 peaks, and were not indexed using *MOSFLM* (gray bars, ∼100 000 patterns). Histograms from *SPIND*-refine and *MOSFLM*-refine fit within the yellow distribution and are omitted for clarity. (*b*) Comparison of indexing rates using *MOSFLM* and *SPIND* with the lattice-refinement option in *indexamajig* enabled and disabled. The lattice-refinement feature requires that more than ten found peaks match their predicted peak positions with a small excitation error (that increases smoothly with resolution) (White, Barty *et al.*, 2016 ▸). Patterns are discarded (not indexed) if this criterion is not met. This contributes to the abrupt cut in indexing rate from using *SPIND*-norefine to *SPIND*-refine since this data set consists of a significant portion of patterns with few peaks (fewer than five).

To understand what caused the 3% low indexing rate, statistics including peak intensity and number of peaks per pattern were obtained for all the crystal hits. A strong correlation between the number of peaks per pattern and the indexing rate was found as shown in Fig. 7 ▸(*a*). For patterns that consist of more than 100 peaks, the indexing rate is above ∼30% (*CrystFEL* 0.6.2), while it drops significantly for the patterns with fewer peaks. Furthermore, as shown by the distribution of number of peaks per pattern (Fig. 7 ▸ *a*), a large portion of the patterns that are identified as crystal hits consist of only ten to 30 peaks. This ineffectiveness and low efficiency in auto-indexing the patterns with a small number of peaks led to the low indexing rate of 3%. To improve the indexing rate for patterns that consist of few peaks, the *SPIND* algorithm was applied to this data set. The indexing rates from all indexing methods are summarized and compared in Fig. 7 ▸(*b*). The *SPIND* algorithm increased the indexing rate slightly, to 4% if using the refinement feature in *indexamajig* (labeled *SPIND*-refine in the figures), and to 54% without the refinement feature. *MOSFLM* indexed 3.1% of the patterns using refinement and 3.5% without the refinement step. *SPIND* orientation solutions were chosen based on scoring the candidate orientations as described in the *Methods* [step (i)[Other li1i]].

**Figure 8:**
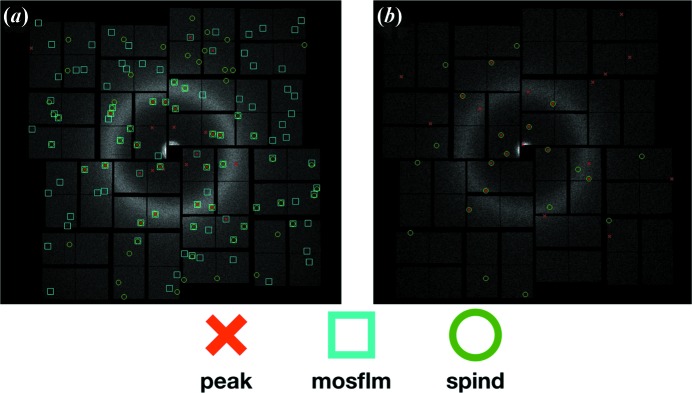
Representative indexed diffraction patterns from the ClR data set, recorded on the CSPAD. (*a*) indexable by both *MOSFLM* and *SPIND*, (*b*) indexable only by *SPIND*. Identified peaks are marked by red crosses, and peak positions predicted from the orientation matrix given by *MOSFLM* and *SPIND* are marked with cyan and green circles, respectively. The overlapping cyan and green circles in (*a*) correspond to the same Miller indices, thus confirming the consistency of the indexing results between *SPIND* and *MOSFLM*.

Lattice and orientation refinement in *indexamajig* requires that more than nine peaks match the predicted peak positions well, from the lowest resolution, with a smooth gradient in excitation error for later refinement. Patterns are regarded as unindexed if this criterion is not met. This contributes to the abrupt reduction in indexing rate when the refinement is included in the analysis of the data set since it consists of a significant portion of patterns with few peaks. Fig. 8 ▸(*a*) shows a representative diffraction pattern that is successfully indexed by both *MOSFLM* and *SPIND*. Almost all patterns indexed by *MOSFLM* were also indexed by *SPIND* (with consistent crystal orientations). Patterns that *MOSFLM* could index but *SPIND* failed to index were found to be multi-crystal patterns. *SPIND* indexed more patterns that were not indexed using *MOSFLM*, and an example is shown in Fig. 8 ▸(*b*). It should be noted that the pattern in Fig. 8 ▸(*b*) has fewer peaks than that in Fig. 8 ▸(*a*). This observation is consistent with the correlation between indexing rate and number of peaks per pattern identified in Fig. 7 ▸(*a*), and validates the capability and effectiveness of the *SPIND* algorithm in indexing patterns with fewer peaks in this data set.

**Figure 9:**
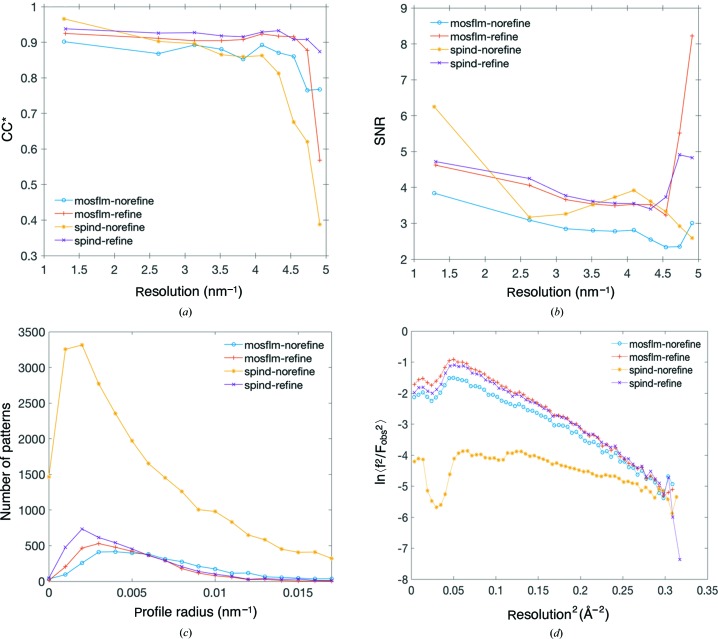
Figures-of-merit for the ClR data set indexed with various indexing algorithms. (*a*) CC*, (*b*) SNR, (*c*) reflection profile radii and (*d*) Wilson plots. The keywords ‘refine’ and ‘norefine’ represent the on and off status of the lattice-refinement option in *indexamajig* of *CrystFEL* in the indexing process. *SPIND*-refine has better figures of merit for this data set than the other methods. Small modal reflection profile radii indicate that orientation determined by *SPIND* is often more accurate than *MOSFLM* with and without orientation refinement.

The ClR data sets were merged with *partialator* (version 0.6.3), excluding reflections with pixel values > 13 200 ADU, push-res = 1.0, with three iterations of scaling and no partiality refinement, resulting in high SNR and CC^*^, but limited completeness at high resolution (making the CC* and SNR misleading in those resolution bins), as shown in Figs. 9 ▸ and S5 in the Supporting information.

**Figure 10:**
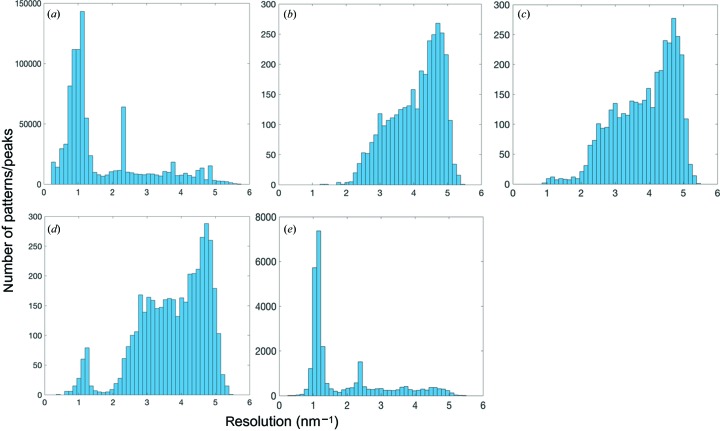
Resolution histograms for the ClR data set. (*a*) Resolution distribution of found peaks for all crystal hits, and distributions of apparent diffraction resolution determined by *indexamajig* after indexing by (*b*) *MOSFLM*-refine, (*c*) *MOSFLM*-norefine, (*d*) *SPIND*-refine and (*e*) *SPIND*-norefine. The additional patterns indexed by *SPIND* are mostly in the lower-resolution region (around 1 nm^−1^) which is consistent with the resolution distribution of the found peaks.

The Wilson plots are linear to ∼2 Å for all indexing methods (Fig. 9 ▸ *d*). *SPIND*-refine performed the best overall, with a higher indexing rate, slightly higher SNR, CC^*^ and smaller modal reflection profile radius (Fig. 9 ▸). The inclusion of a large number of low-resolution patterns by *SPIND*-norefine yielded a higher SNR and CC^*^ in the lowest resolution shell, but performed worse at medium and high resolution. To understand the contrasted behavior between low- and high-resolution ranges, the histograms of the apparent diffraction resolution that is estimated by *CrystFEL* per pattern for different indexing methods are compared with the resolution histogram of the found peaks (Fig. 10 ▸). The resolution distributions of the patterns indexed by *SPIND* are consistent with that of all found peaks, showing clustering in both low- and high-resolution ranges, while other indexing methods favor more high-resolution patterns. The peak in the low resolution range tails at about 1.2 nm^−1^ in Figs. 10 ▸(*d*) and 10(*e*) explains the significant increase in SNR in the lowest resolution bin (Fig. 9 ▸ *b*). The kink in the *SPIND*-norefine Wilson plot (Fig. 9 ▸ *d*, yellow line) around 0.15 Å^−2^ is probably caused by the imperfect scaling of intensities from two distinct crystal batches, judging by their diffraction resolution (see resolution histograms in Fig. 10*e* ▸), which is likely to be indicative of anisomorphism between the two crystallization batches. The accuracy or orientations determined by *SPIND*-norefine can be inferred by the small reflection profile radii, showing again that *SPIND*, even without orientation refinement, did determine accurate orientations for the majority of the patterns.

The refinement module in *indexamajig* optimizes and refines the lattice constants and crystal orientation for each indexed pattern by minimizing the residuals between the experimental peak positions and the peak positions that are predicted from the orientation matrix given by the auto-indexer. Therefore, for *SPIND*-norefine and the 3-ring integration method (White *et al.*, 2012 ▸) that is usually adopted for intensity integration using *indexamajig*, the background, signal and noise are incorrectly estimated since the predicted peak positions may not match the observed peak positions in the higher-resolution range without lattice refinement. The addition of an orientation refinement module requiring fewer peaks will improve merging statistics from *SPIND* at higher resolution than demonstrated here, if not limited by crystal quality.

### 3.4. Software availability, usage and performance   

**Figure 11:**
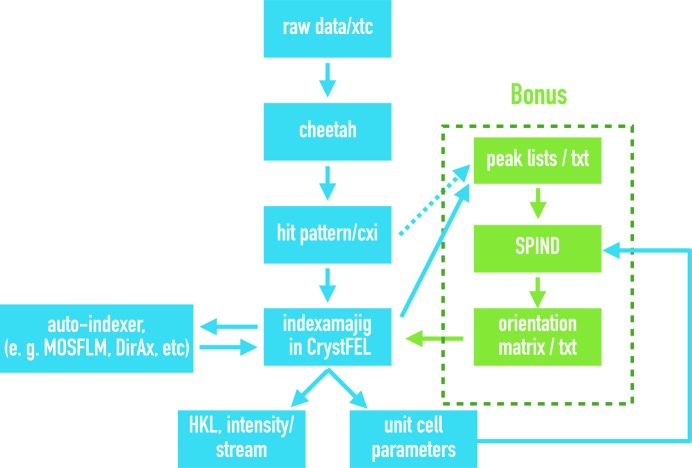
Schematic SFX data-analysis pipeline integrating *SPIND* to *CrystFEL*.

The algorithm development and prototype test of *SPIND* were first conducted in *MATLAB*. For compatibility and portability, *SPIND* includes recently updated features designed for protein serial crystallography and is publicly available under the GNU General Public License from https://github.com/LiuLab-CSRC/SPIND. For the user’s convenience, *CrystFEL*, with *SPIND* integrated as an alternative indexing module callable from *indexamajig,* is also available from the repository. The *CrystFEL* data-analysis pipeline incorporating *SPIND* is shown in Fig. 11 ▸. It is recommended to use *MOSFLM* and *DirAx* *etc*. for auto-indexing first and then apply *SPIND* to improve the indexing rate based on the reference unit cell given by previous indexers.

The computation time and required memory are mainly determined by two factors. (1) the length of the structure-factor list used to generate the reference table. The computation time and required memory roughly follows *N* ^2^, where *N* denotes the number of Bragg reflections included in the reference structure-factor list. (2) Error-tolerance threshold in the vector-searching process (see the *Methods* section[Sec sec2]). Larger threshold values generally lead to longer computation time. Auto-indexing using *SPIND* can be very time and memory demanding for protein crystallography because of the large number of Bragg reflections used in the reference list. In principle, the reference structure-factor list is generated only for the resolution range where most of the experimental peaks fall, to minimize the memory needs and computation time. In addition, the vector-tolerance threshold can be set to be small first, and then be adjusted to be larger to increase the indexing rate with a longer but reasonable computation time. *SPIND* also supports parallel computation using multiple CPUs. As an example, the computation time to auto-index a subset of 4962 patterns from the DOR SFX data set was around 12 core hours (Intel Xeon E5-2680 v3 at 2.5 GHz).

## 4. Conclusions   

Diffraction patterns that consist of a small number of peaks often take up a significant portion of the whole data set in serial protein crystallography. The insufficient number of peaks along with the poor diffraction quality make these patterns difficult to analyze using the Fourier transform-based algorithms. In order to utilize these data and increase the data efficiency, we have developed a new auto-indexing algorithm, *SPIND*. It is based on identifying the Miller indices of five peaks chosen from each pattern by comparison with the reference unit cell.

The algorithm was tested first using simulated diffraction data from I3C microcrystals in random orientations and with random fluctuations in lattice constants. All 400 simulated sparse I3C patterns were auto-indexed successfully using only three to five Bragg peaks per pattern. Each pattern was auto-indexed in milliseconds, to an accuracy of 0.1° in the Euler angle defining the crystal orientation. This shows the robustness of the algorithm to lattice inhomogeneity and distortions. *SPIND* was then shown to perform as well as established auto-indexers *MOSFLM* and *DirAx*, slightly improving the indexing of SFX data from microcrystals of a GPCR complex (CXIDB ID 40). The SNR and self-consistency figures of merit all slightly improved over the whole resolution range by using *SPIND* with orientation refinement in *indexamajig*. Finally, *SPIND* was used to improve a data set from ClR crystals. The indexing rate using *MOSFLM* was around 3% because of the insufficient number of peaks in most of the patterns, along with poor diffraction quality. Even without orientation refinement in *indexamajig* (which requires at least ten Bragg spots), *SPIND* improved the merged data quality in the lower resolution range by indexing additional patterns of low resolution. However, the overall resolution limit of the whole data set was ultimately limited by the low diffraction quality of the crystals.

These results demonstrate that *SPIND* can index serial microcrystal diffraction patterns with very few Bragg reflections (*e.g.* inorganic microcrystals with small unit cells), and improve the quality of membrane-protein SFX data. The growing adoption of serial crystallography methods at synchrotron beamlines, using continuous injection of a stream of microcrystals across the beam and fast recording (Standfuss & Spence, 2017 ▸), as well as micro-electron diffraction from inorganic and macromolecular microcrystals, will also benefit from the algorithm described here. *SPIND* is actively being developed to include an optimized search algorithm, multi-crystal indexing, orientation refinement and more features for improving protein SFX indexing. It is written in Python and is publicly available from https://github.com/LiuLab-CSRC/SPIND.

## Supplementary Material

Supplementary Figures. Processing details of the representative data sets.. DOI: 10.1107/S2052252518014951/cw5018sup1.pdf

## Figures and Tables

**Table 1:** Parameters for hit finding of DOR data set using *Cheetah* (Fenalti *et al.*, 2015 ▸)

No. of frames collected	Minimum no. of peaks per frame	No. of pixels per peak	Peak intensity threshold (ADU)	SNR threshold	Peak search region	Peak finder algorithm	No. of hits found (hit rate %)
1967539	15	2 – 40	40	4	70–700 pixels from center	8	125458 (5.9)

**Table 2:** Parameters for auto-indexing of DOR SFX data using *indexamajig*, calling *DirAx*, *MOSFLM* and *SPIND* as subroutines

Indexer	Reference cell	*indexamajig* orientation refinement	Unit-cell tolerance (vector-search tolerance in *SPIND*)	Radii of peak integration and background rings (pixels)	No. of indexed patterns (indexing rate %)
*DirAx*	Yes	Yes	5%, 1.5°	3, 4, 5	65015 (51.8)
*MOSFLM*	Yes	Yes	5%, 1.5°	3, 4, 5	57687 (45.9)
*MOSFLM*	No	Yes	5%, 1.5°	3, 4, 5	57413 (45.7)
*SPIND*	Yes	Yes	5%, 1.5° (3 × 10^7^ m^−1^, 3°, reference resolution > 8 Å)	3, 4, 5	67204 (53.5)
*SPIND*	Yes	No	5%, 1.5° (3 × 10^7^ m^−1^, 3°, reference resolution > 8 Å)	3, 4, 5	118514 (94.4)

**Table 3:** Merging statistics from DOR SFX data using *partialator* The values in the parentheses are for the highest-resolution shell (2.8–2.7 Å)

Indexing method	No. of patterns used	No. of crystals merged	Resolution range (Å)	*R*_split_ (%)	CC*	SNR
*DirAx*-refine	65015	64918	34.3–2.7	11.9	0.9982 (0.7069)	6.3
*MOSFLM*-refine	57687	57597	34.3–2.7	12.6	0.9979 (0.6803)	6.1
*MOSFLM*-nolatt-refine	57413	57309	34.3–2.7	12.1	0.9980 (0.6704)	6.2
*SPIND*-refine	67204	67067	34.3–2.7	11.7	0.9981 (0.6992)	6.5
*SPIND*-norefine	118514	116198	34.3–2.7	14.7	0.9973 (0.5978)	5.8
