# Supplementary material for: SPIND: a reference-based auto-indexing algorithm for sparse serial crystallography data
Source: IUCrJ. 2019 Jan 1;6(Pt 1):72–84. doi: 10.1107/S2052252518014951 (PMC6327178; doi:10.1107/S2052252518014951)
Supplement: Supplementary file 1 [file m-06-00072-sup1.pdf]

IUCrJ

Volume 6 (2019)

Supporting information for article:

SPIND: a reference-based auto-indexing algorithm for sparse serial crystallography data

Chufeng Li, Xuanxuan Li, Richard Kirian, John C. H. Spence, Haiguang Liu and Nadia A. Zatsepin

Indexing SFX data from a G protein-coupled receptor complex

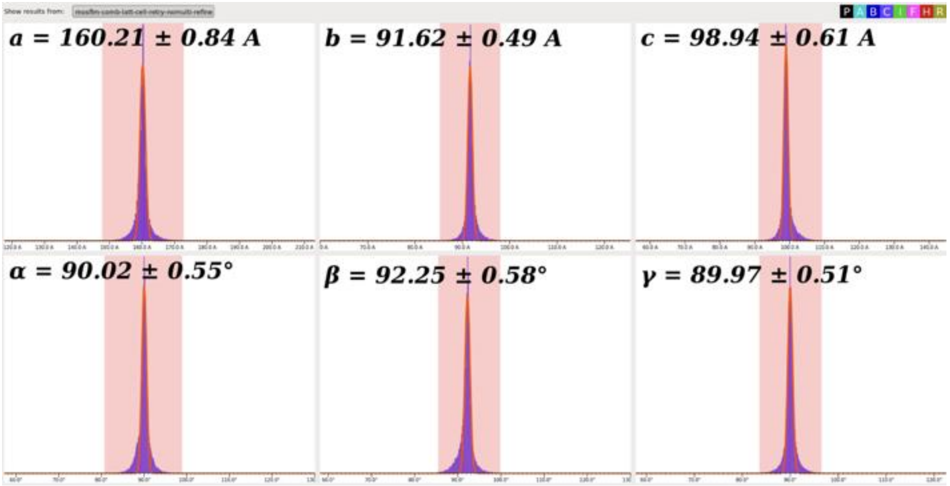

Figure S1. Updated unit cell for DOR, slightly larger than published values (Fenalti et al. 2014 and White, Barty et al. 2016). These updated cell parameters were used for all indexing methods in this paper.

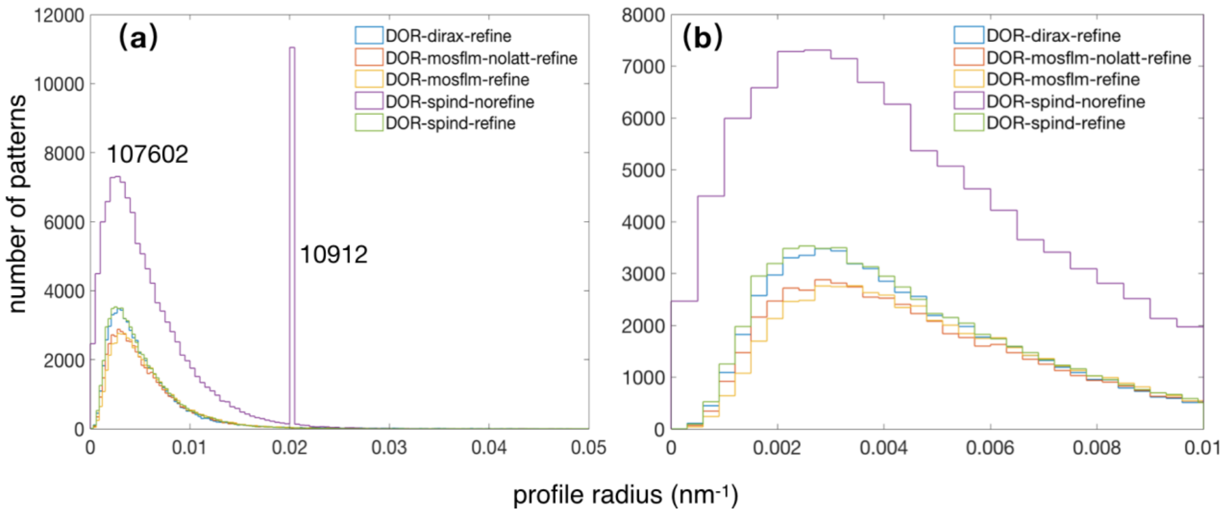

Figure S2. Histogram of profile radius estimated by *indexamajig* after indexing for indexing methods "DirAx-refine", "MOSFLM-nolatt-refine", "MOSFLM-refine", "SPIND-refine", and "SPIND-norefine". (b) is shows the enlarged view of (a). The profile radius value of 0.02 nm-1 was assigned to 10912 patterns, 9.2% of the patterns indexed by SPIND-norefine in total. These may correspond to the indexing solutions that do not give good match between the found and predicted peaks and hence failed the profile radius optimization without lattice and orientation refinement. The distribution of the profile radius for the rest of the patterns (85.7% of the total crystal hits) indexed by SPIND-norefine is consistent with

those from other indexing methods. This indicates the need of a lattice refinement algorithm for such sparse patterns.

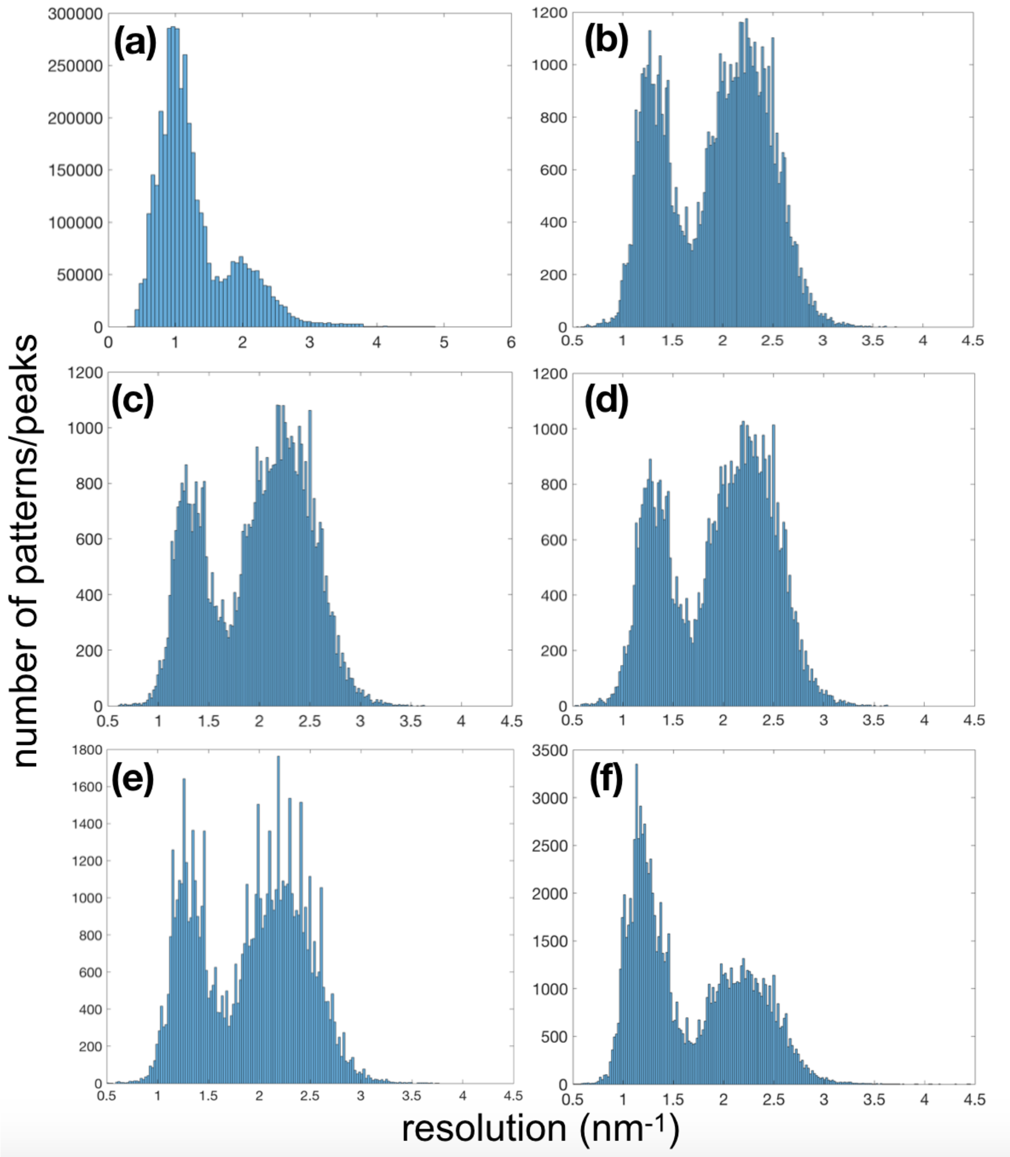

Figure S3. Resolution histograms for DOR datasets (a) resolution distribution based on found peaks for all crystal hits. The apparent diffraction resolution determined by *indexamajig* after indexing for indexing method (b) DirAx-refine, (c) MOSFLM-nolatt-refine, (d) MOSFLM-refine, (e) SPIND-refine, and (f) SPIND-norefine. The distribution of pattern resolution determined by *indexamajig* is consistent with that of the resolution distribution of all found peaks in this data set. SPIND indexed a larger portion of patterns in the lower resolution range compared to other indexing methods.

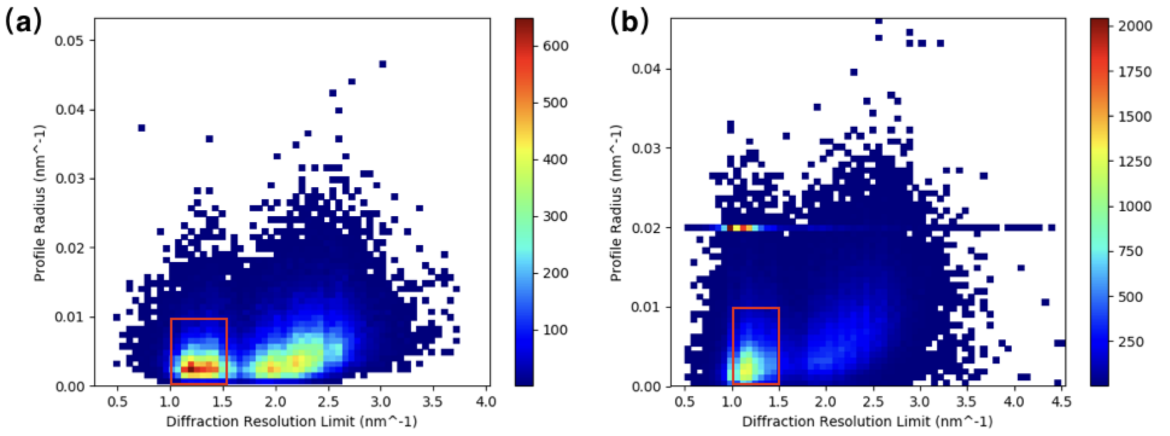

Figure S4. Histograms of diffraction resolution limit vs Bragg profile radius (indicating accuracy of determined orientations) for the DOR datasets using (a) SPIND-refine and (b) SPIND-norefine. When *indexamajig* does not have a sufficient number of peaks matched, the Bragg profile radius is not updated from the default 0.02e9 nm^{-1}, as visible in (b) and Figure S2(a). The number of patterns in the red boxes (resolution between 1.0 and 1.5 nm^{-1}, and profile radii between 0 and 0.01 nm^{-1}) is (a) 18,874 out of a total 67,204 indexed (28%) and (b) 45,862 out of a total 125,458 indexed (36%), which highlights that despite the lack of orientation refinement in SPIND-norefine (b), a larger total number of patterns was accurately indexed by SPIND.

Indexing SFX data from chloride ion-pumping rhodopsin (ClR) microcrystals

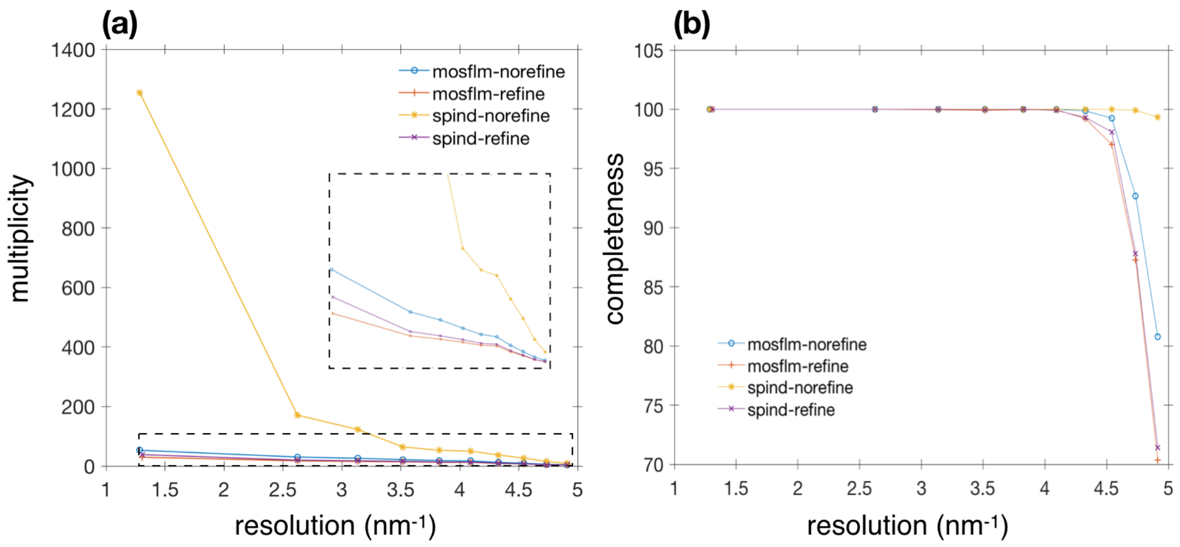

Figure S5. Multiplicity (a) and completeness (b) in resolution bins for the ClR dataset. Multiplicity increases with increased Bragg profile radii, and completeness is solely based on multiplicity (without regard for SNR) so these may be overestimated.
